# A research agenda on oral health care as a boundary object that unites the perspectives of patients and practitioners

**DOI:** 10.1111/hex.13310

**Published:** 2021-07-27

**Authors:** Femke Hilverda, Puck van der Wouden, Geert J. M. G. van der Heijden, Carina A. C. M. Pittens

**Affiliations:** ^1^ Athena Institute Faculty of Science VU University Amsterdam Amsterdam The Netherlands; ^2^ Department of Socio‐Medical Sciences, Erasmus School of Health Policy and Management Erasmus University Rotterdam Rotterdam The Netherlands; ^3^ Department of Oral Public Health Academic Centre for Dentistry Amsterdam VU University Amsterdam Amsterdam and University of Amsterdam Amsterdam The Netherlands; ^4^ Amsterdam Public Health Institute Amsterdam The Netherlands

**Keywords:** boundary‐work theory, oral health care, patient perspective, research agenda setting

## Abstract

**Context:**

A research agenda for oral health care was established in the Netherlands using the Dialogue Model. This project served as a case study in which we applied boundary‐work theory as a framework to understand boundaries (ie demarcations) between and within groups, and how these boundaries can be overcome.

**Objective:**

To gain insights into the boundaries encountered when setting a research agenda, we analysed how this agenda served as a boundary object (ie circumstances, situations or material that connect actor groups and allow boundary crossing) that facilitated crossing boundaries and uniting the perspectives of patients and practitioners.

**Methods:**

We used a thematic approach to analyse researchers' observations, meeting materials, emails, interviews with patients (n = 11) and a survey among patients and practitioners (n = 18).

**Results:**

Setting the research agenda helped to cross boundaries in oral health care, which demonstrates its role as a boundary object. First, this made it possible to integrate research topics representing the perspectives and priorities of all patients and also to unite those perspectives. It was essential to involve practitioners at an early stage of the project so that they could better accept the patients' perspectives. This resulted in support for an integrated research agenda, which facilitated the crossing of boundaries.

**Conclusions:**

The research agenda‐setting project was found to serve as a boundary object in uniting the perspectives and priorities of patients and practitioners.

**Patient contribution:**

Patient involvement in this case study was structured in the process of research agenda setting using the Dialogue Model.

## 1 INTRODUCTION

A research agenda—a list of prioritized research topics—is an essential tool for providing directions for future research. At the same time, it also supports funding agencies and research institutes in their programming and implementation of health‐care research.^1^ Traditionally, agenda setting for health‐care research has been driven mainly by those involved in conducting research, notably researchers and funding agencies. Patients are rarely involved in initiating health‐care research,^2^, ^3^ and as such, agenda setting in health care can be viewed as an approach that is driven by interest and supply. From 2000 onwards, patient involvement in medical research has gradually increased.^3^, ^4^

Involving patients in research agenda setting allows them to represent their preferences and experiences as the end‐users of health care, and taps into their views, needs and perspectives in daily life and health‐care practice. As such, it is assumed that patient involvement will result in research that caters more specifically to their needs because they provide unique insights as experts in their own right.^5^, ^6^, ^7^, ^8^, ^9^, ^10^ Moreover, it has been argued that it is a patient's right to be involved in issues that affect them personally.^11^

The involvement of patients in research agenda setting has shown to enhance their empowerment^12^ and may facilitate acceptance of a research agenda.^13^, ^14^ Patient involvement also adds to the legitimacy of research policies and decision‐making processes^10^, ^15^, ^16^ by stimulating research topics that patients consider important.

Involving patients is, however, not self‐evident and presents various challenges. Elberse and colleagues^7^ identified ways in which patients were excluded from setting research agendas. Exclusion can occur when patients' input is dismissed or regarded as irrelevant or when researchers and practitioners use too much jargon. Apart from their exclusion, involving patients in health‐care research involves addressing other challenges: (a) policies on involving patients as co‐creators of research can be ambiguous; (b) identifying the target group of patients and how they should be approached is not straightforward; and (c) researchers and other stakeholders do not value patient involvement.^17^

While patient involvement is becoming common in research on health‐care issues, this often lags behind in domains in which a patient group is not easily defined, such as oral health care.^7^, ^18^, ^19^ To address this, a research agenda for oral health care was established in the Netherlands. The two most important stakeholder groups for this research agenda, patients and practitioners, were consulted. The oral health‐care practitioners included general dentists, specialized dentists, oral and maxillofacial surgeons, orthodontists, dental hygienists and (prosthetic) dental technicians. The patients consulted were high‐risk groups in relation to oral health‐care issues (patients with diabetes, lung problems, cardiovascular diseases, depression, or rheumatism).

In this study, we explored boundaries in relation to the involvement of and interaction between patients and practitioners in the process of research agenda setting using boundary‐work theory as a theoretical framework.

### 1.1 Boundary‐work theory

Boundary‐work theory^20^, ^21^ was used to guide this study. Boundary‐work theory originates from sociology and was used to explain boundary work in the domain of science by examining social boundaries that scientists set to distinguish science and its products from non‐scientific activities.^20^ Other social domains, such as educational sciences^22^ and health‐care practice,^19^, ^23^, ^24^ later adopted the concept of boundaries.

Boundaries can be viewed as dissimilarities between objects, individuals, ideas or actions that create distinctive categories among these.^20^, ^25^, ^26^ They are demarcations of professions, and within that, demarcations of disciplines, specialties, theoretical orientations or interests within a profession may emerge.^20^ Such demarcations manifest as socially constructed boundaries of a social or symbolic nature.^25^ They often contribute to the autonomy and authority of professions and disciplines^27^ and play a role in inter‐professional interactions. In this way, a boundary is often perceived as an obstacle that persons or groups experience that hinders or precludes their communication or collaboration or both. Such boundary separates them, but can be crossed when they are brought together to engage in resolving this hindrance. For example, an obstacle that could occur between patients and practitioners could be a lack of understanding for each other's perspective. This lack of understanding enhances the difference between the distinctive categories of patients and practitioners and in this way acts as a boundary. Individuals or groups are continually able to define, sharpen or soften these boundaries in an attempt to maintain or strengthen their autonomy, authority and interests. Such action is referred to as boundary work.^20^, ^21^

Three types of boundary work can be distinguished: (a) *protection*, (b) *expulsion*, and (c) *expansion*.^21^ Protection is directed at maintaining the existing boundaries. Expulsion sharpens boundaries through the monopolization of professional authority, resources and results by the rejection of other individuals, while expansion implies crossing or entering different categories and creating a new, broader perspective. The Dialogue Model is based on this latter principle of boundary work.^19^

In relation to expansion boundary work, *boundary crossing* is especially relevant. Boundary crossing was established by Suchman^28^ (p. 25), who defined it as ‘to enter onto territory in which we are unfamiliar’. Engeström et al^29^ (p. 319) defined boundary crossing as ‘negotiating and combining ingredients from different contexts to achieve hybrid situations’. Crossing boundaries therefore describes the process of entering different categories by negotiation and interaction, which leads to the transformation of categories and the creation of a new, broader perspective. In relation to oral health care, one could think about practitioners going beyond their focus on technical procedural aspects of oral health care. By crossing the boundary to reach patients and using insights from patients' perspective, a more complete picture about oral health‐care arises.

To facilitate boundary crossing, the identification or development and use of boundary objects are an essential process. Boundary objects were defined by Star & Griesemer^30^ (p. 393) as ‘…objects which both inhabit several intersecting social worlds and satisfy the (informational) requirement of each of them’. In this way, boundary objects fulfil a symbolic role as a bridge because they connect actor groups and allow them to cross a boundary.^30^ Boundary objects should allow the involved actor groups, on the one hand, to adapt to the conditions and needs of all, and, on the other hand, to establish a common identity.^30^ A boundary can thus be crossed when persons or groups engage to jointly remove boundaries or when a boundary object initiates communication or collaboration. For example, the research agenda might act as a boundary object between patients and practitioners, because setting the research agenda helps to understand each other's perspective better. In this way, the possible boundary of lack of understanding is crossed. In addition, the research agenda can thus be seen as a communication tool which allows people from different groups to talk to each other.

## 2 METHODS

To gain insight into boundaries encountered when setting a research agenda and how the research agenda might function as a boundary object, we evaluate the process of developing a research agenda in the area of oral health care as a case study and reflect on how each method used in this process helped to move towards the development of the research agenda.

### 2.1 Case description

In the Netherlands, a research agenda for oral health care was constructed with input from oral health‐care patients and practitioners using the Dialogue Model.^16^ To accomplish this, a staged approach was used. First, a practitioners' research topic list was created in 2016 and 2017. Next, a patients' research topic list was constructed in 2018. Finally, in 2019, the perspectives of patients and practitioners were combined in a consensus session to establish a shared research agenda. Box 1 describes the stages of the Dialogue Model and corresponding research activities, while Box 2 presents the shared research agenda. The construction of the research agenda is discussed in detail elsewhere (https://www.mondzorg2020.nl/).

BOX 1 **Phases of the Dialogue Model and corresponding research activities**

The Dialogue Model, consisting of six phases, was developed and validated to structure the process of patient involvement in research agenda setting in the Netherlands.^16^ Central to the Dialogue Model is the recognition of stakeholders' different perspectives, which stimulates direct interaction and the co‐production of knowledge.^31^ The Dialogue Model is a multi‐phased participatory approach that is used for setting a research agenda with multiple stakeholders including patients. It is based on six underlying key principles specifying how the process needs to be conducted: (a) active involvement of patients; (b) conductive social conditions; (c) respect for patients' experiential knowledge; (d) on‐going dialogue, paying particular attention to reflexive learning processes; (e) emergent and flexible design; and (f) impartial process facilitation. The Dialogue Model operationalizes *consultation* and *collaboration* among multiple stakeholders and emphasizes learning processes by stimulating dialogue between stakeholders.^16^ The model has been applied in many agenda‐setting processes.^7^, ^12^ The model has an emergent design in which activities are structured in six phases: exploration, consultation, prioritization, integration, programming and implementation. Programming (phase 5) and implementation (phase 6) were beyond the scope of this research. In this project, we focused on the first four phases:

1. *Exploration:* In this phase, the first insights into the problem are gained and stakeholders' various needs and wishes in relation to the required process are identified (*identification of conductive social conditions*). During the exploration phase, a professionals' stakeholder meeting was organized to gain support for the project. Participants (n = 25) included a broad variety of opinion leaders in the dental health‐care field (n = 15), researchers (n = 5), research policymakers (n = 3), a representative from the dental industry and an expert in patient involvement. In addition, five patients' organizations were approached to explore the feasibility of the project and to collaborate in recruiting patients.
2. *Consultation:* During this phase, the goal is to identify separate research topics for each stakeholder group to ensure enclave deliberation.^32^ Oral health‐care practitioners were asked via a questionnaire (n = 210) to name subjects for future research. These were grouped into themes and translated into research topics. A different approach was used for patients. The barriers that patients experience in their daily lives regarding oral health care were mapped during focus groups. A total of four focus groups took place with high‐risk groups (patients with diabetes [n = 7], lung problems [n = 6], cardiovascular diseases [n = 6], or rheumatism [n = 8). One focus group was arranged for each high‐risk group. It was difficult to recruit patients with depression to participate in a broad discussion, so to take account of their perspective on barriers, it was therefore decided to conduct interviews (n = 3) separately with this particular patient group. Subsequently, the barriers were grouped into themes and translated into research topics by the researchers.
3. *Prioritization:* In this phase, the goal is to prioritize research themes for each group separately. In a second survey, oral health‐care practitioners (n = 235) prioritized the research topics extracted from the first survey, which resulted in a top‐10 list for future research. Regarding patients, the research topics extracted from the focus groups and interviews were checked for endorsement and prioritized in a survey study among a larger sample of patients (n = 1495) to establish their top‐10 topics for future research.
4. *Integration:* During this phase, the goal is to integrate the list of research topics of practitioners with the research topic list of patients via dialogue. To create a fruitful consensus meeting, values like respect, tolerance, willingness to listen, openness and inclusion are vital to both stakeholder groups and researchers.^7^ Integration was accomplished via a consensus session in which the perspective of practitioners (n = 10) and the perspective of patients (n = 11) were combined to establish a shared research agenda. Practitioners included general dentists, specialized dentists, dental hygienists and a dental technician. We approached patients who had indicated they wanted to participate in the consensus meeting in the prioritization survey. We invited patients with a diverse background in terms of diseases, whose three priority topics were included in the top 10. The top‐10 topics of patients and practitioners were used as a starting point for discussion. At the end of this meeting, a final voting took place in which three topics were selected per participant. The shared research agenda consisted of 8 topics.

BOX 2 **Research agenda**

A shared agenda with eight priority topics for future oral health‐care research was established, containing eight topics prioritized by patients and practitioners. The shared research agenda consists of five topics originating from the patients' topic list (#3, #4, #5, #6 and #8), two topics originating from the practitioners' topic list (#1, #7) and one topic that was found on both lists (#2).

Topics on the shared research agenda:

1. How can we change behaviour to improve oral health care?
2. What is the relation between oral health care and (medical and psychological) conditions?
3. How can we increase the involvement of oral health‐care practitioners with other health‐care practitioners?
4. Research on how oral health care can be adapted for patients with chronic diseases
5. Research on how insurance for oral health care can be added to basic health insurance
6. Research on how the knowledge of (oral) health‐care practitioners can be increased beyond their expertise
7. Oral health care for elderly people: what are the consequences of treatment and treatment planning?
8. How can shared decision making in oral health care be implemented?

### 2.2 Data collection and participants

The activities that were carried out to establish the research agenda are presented in Box 1. In addition to these activities, we also took steps to evaluate the process and to identify boundaries encountered, how they were crossed and to what extent the research agenda functioned as a boundary object. These evaluation actions served as our central data gathering methods and comprised the following data sources:

- *Researchers*' *observations during each phase of the Dialogue Model:* Observations were made during all the activities that were undertaken to create the research agenda (Box 1). During these activities, the researchers observed the participants' behaviours, their input into the research agenda and their behaviour towards each other. In addition, possible boundaries of setting the research agenda and how they were addressed were observed.
- *Documents related to stakeholder meetings:* During the project, three stakeholder meetings took place (see Table 1). These meetings were attended by approximately 30 participants, of which half were oral health‐care practitioners. Other stakeholders were patient representatives, researchers, medical practitioners, policymakers, representatives from the dental industry and research funders. Documents related to stakeholder meetings, such as reports, emails and notes from group discussions, were collected and studied.
- *Emails received from patient organizations (*n* = 5):* Responses from patients' organizations were filed and studied to map the boundaries encountered.
- *Interviews with patients:* After the four focus groups that were conducted in the consultation phase (see Box 1), we interviewed several patients who participated in them. During these semi‐structured face‐to‐face interviews (n = 11), the emphasis was on the experience of meaningful involvement and not on the outcomes of the focus groups.
- *Questionnaire after consensus session:* After the consensus session (Box 1), participants (n = 21) received a short questionnaire (see Appendix A) on how they had experienced this session and were asked how they thought the session could be improved. The questionnaire was filled out by 18 participants.

**Table 1:** Number of participants and main results of the stakeholder meetings

	Meeting 1: 2015	Meeting 2: 2017	Meeting 3: 2018
Total number of participants (oral health‐care practitioners/other stakeholders)	25 (15/10)	31 (16/15)	32 (14/18)
Main goals of the meeting	Introduce project Consultation on method of topic collection among practitioners Support and commitment for the project	Discuss results of topic collection Consultation on method of prioritization among practitioners Introduction of patient engagement during project Support and commitment for the project	Discuss results of prioritization and reach consensus on top 10 Further consideration of patient engagement in project Support and commitment for the project
Main results	Consensus about the target group was reached: the full range of oral health‐care practitioners should be included (eg dental specialists, dental hygienists) Recommendation: development of an online survey to identify and collect topics	The final research agenda will have to include an equal contribution from patients and practitioners Consensus on the suggested method for prioritization was reached	Agreement on the top‐10 research topics was reached among practitioners

### 2.3 Data analysis

All interviews were audio‐taped and transcribed verbatim in Dutch. Observations, document analysis and conversations within the project team and with others (in person or via email) were noted in detail in the researchers' logbook.

The interview transcripts and logbook (including observational data) and questionnaire on points for improvements after the consensus meeting were analysed using thematic content analysis.^33^ Thematic analysis is a method to identify, analyse and report underlying patterns and themes. The data were analysed by applying the theory of boundary work; anticipated and encountered boundaries formed the basis of the coding. In this process, information from the transcripts and logbook was complemented with improvements points gathered in the questionnaire to extract information on boundaries encountered in the process of establishing a research agenda. General boundaries were defined, as well as boundaries within stakeholder groups and between stakeholders. For example, we had expected to find a boundary between patients and practitioners, but analysis of the data also revealed a boundary among groups of patients, arising from the diversity of oral health‐care patients. The strategies that were used to cross boundaries were examined and the role of the research agenda as a boundary object was also investigated. The coding process was performed in an iterative manner by authors FH and PvdW with assistance from CP. Coders checked each other's coding and discussed differences until consensus was reached. In addition, coding was discussed within the research team, regularly.

### 2.4 Ethical consideration

All participants who took part in activities related to setting the research agenda as well as activities that were carried out to evaluate the process received written and verbal information beforehand about the goal of this research project. It was explained to them that participation was voluntary and that they were able to withdraw at any time without any consequences.

Approval of Ethics Committee of Academic Centre for Dentistry Amsterdam was provided on 15 February 2018, with the document number: 2018009. During the collection and handling of data, the applicable privacy and data protection regulations were followed so that data could not be traced back to individuals.

## 3 RESULTS

Several boundaries were encountered during the research agenda‐setting process. We differentiated between general boundaries, such as possible lack of support, and boundaries related to specific stakeholders, such as the difficulty in reaching oral health‐care patients. In addition, boundaries between and within groups of patients and practitioners were distinguished. For each boundary, we describe which strategies were used to overcome them and the role of the research agenda in this process. An overview of the results is presented in Table 2.

**Table 2:** Boundaries, strategies and the role of the research agenda

Stakeholders	Boundary	Strategy/reaction	Role of research agenda
General	Possible lack of support for the research agenda	Engagement of stakeholders from the start of the project and during the entire process (eg via structural stakeholder meetings during project)	Setting the research agenda created the involvement of different stakeholders in the agenda‐setting process
	Reach representative group of patients and practitioners	Inclusion and transparency; Dialogue Method	Creating a research agenda via the Dialogue Method ensured representation of patients and practitioners
Patients	Difficulty in reaching oral health‐care patients because they are not a well‐organized patient group	Focus groups with patients with chronic diseases (for which a patient organization or patient platform exists)	Research agenda setting created awareness of oral health‐care issues among patients (with and without chronic diseases)
	The perception of patient organizations that oral health was not a topic of interest for their patients	Bottom‐up recruitment strategies: approaching individuals (via social media or patient meetings) with specific interest in the topic	Awareness is created among patient organizations that oral health‐care problems are important to patients because they influence wellbeing/quality of life
	Diversity of oral health‐care patients	Initial consultation of patients per chronic disease group, where after a survey among broader group of patients was conducted	Research agenda setting stimulated patients to think about a variety of issues related to oral health care. Discussing and recognizing oral health‐care problems made it possible for them to learn from each other
Patients and practitioners	The difference in perspectives and interests of patients and practitioners	Consult each actor group separately, then have a consensus meeting	Creating a research agenda via dialogue ensured that shared topics were prioritized
	Uncertainty about the value of patient involvement	Gradually increase the role of patients in the project: step‐by‐step introduction. Meetings were moderated in a way that meant patient input was secured and valued	Research agenda setting made the patients and practitioners involved realize that patients can supply valuable information from their experiences
Practitioners	Unfamiliarity of oral health‐care practitioners with research agenda setting	Consult patients and practitioners separately and sequentially	Setting the research agenda resulted in the involvement of practitioners in the agenda‐setting process
	Lack of urgency for a research agenda	Emphasis on communication about the project and long‐term benefits for the individual professional as well as the profession	The research agenda created awareness that increased evidence was needed for oral health care
	Practitioners prefer topics that fit their own specialty	Design of the survey: maximum of 2 topics per domain in the top 10	Research agenda setting stimulated practitioners to broaden their focus and to reflect on uncertainties in daily practice

### 3.1 General boundaries

Two general boundaries were encountered. These boundaries were present for patients as well as practitioners. Firstly, in the exploration phase we discovered *a possible lack of support for the research agenda* to be a general boundary. This boundary occurred on the institutional level, meaning that lack of support was expected or found on an organizational policy level rather than individual patients or practitioners expressing lack of support. During the first stakeholder meeting participants mentioned that the profession of oral health care does not value patient input to the same extent as input from practitioners, and thus, little interest in creating a shared agenda was expected. In addition, it became apparent during this phase that patients' organizations did not perceive oral health care to be an important topic for their members. To overcome this experienced lack of support, or even resistance, we stimulated engagement of stakeholders from the start of the process via structural stakeholder meetings during the project. During these meetings, the involvement of different stakeholders in the agenda‐setting process was assured by involving them closely in the research process and hearing their perspective. At the first meeting, stakeholders signed a commitment form, making their commitment to the research agenda‐setting process explicit.

Secondly, we anticipated that it would be challenging to *reach a representative group of patients and practitioners*. Using the Dialogue Method in setting the research agenda allowed for co‐creation and inclusion during the research agenda‐setting process. Creating a research agenda via the Dialogue Method stimulated engagement of patients and practitioners and structured the dialogue about perspectives and priorities among and between them. However, to be able to reach patients and practitioners multiple recruitment strategies might be needed. To ensure representation of both patients and practitioners, continuous active recruitment and involvement were required.

### 3.2 Boundaries in relation to patients

Three boundaries were experienced in relation to patients: (a) the difficulty in reaching oral health‐care patients because they are not a well‐organized patient group, (b) the perception of patient organizations that oral health care was not a topic of interest for their patients, and (c) the diversity of oral health‐care patients that we encountered.

The first boundary encountered in relation to patients included *the difficulty in reaching oral health‐care patients because they are not a well‐organized patient group*. Generally, oral health‐care patients are not recognized as patients and do not recognize themselves as patients. As a consequence, there is no patient organization that represents oral health‐care patients. This complicated the recruitment of oral health‐care patients. During the second stakeholder meeting, participants were consulted about how to establish patient involvement for the research agenda to bypass the lack of organization of patients. During this meeting, the suggestion to focus firstly on patients with chronic diseases was widely supported. There are organizations that represent these patients, such as individuals who suffer from diabetes, and thus are easier to reach. We focused on patients with chronic diseases who have an increased risk of oral health(care) problems because they had diabetes, cardiovascular diseases, depression, rheumatic disorders or a lung disease. This resulted in deciding to organize focus groups for individuals with chronic diseases first. The results from these focus groups were used to design a survey study that was conducted among a broader group of patients. Using this approach, with an enormous response rate, research agenda setting created awareness of oral health‐care issues among a wide range of patients, with and without chronic diseases.

Secondly, during the exploration phase, it became clear that most *patient organizations perceived oral health care not as a topic of interest for their patients*. While the Netherlands Patient Federation, a large Dutch patient organization for patients with a variety of diseases, perceives oral health care as an important topic, most patient organizations did not perceive oral health care to be a problem for their patients, whereas they were familiar with the increased risk and prevalence of oral health‐care issues that concerned their patients. They were unwilling to assist with recruitment. When we contacted these organizations (by email or phone), they explicitly dismissed oral health care as a priority topic for their patients and protected their boundaries by refusing to help. One of the organizations' contact persons replied: ‘After internal discussion, I confirm that this topic has no priority for us and that there are other projects that are closer to us…’. This feeling must have been shared by the other organizations, because similar responses were obtained. We responded to this boundary by using different recruitment strategies. We first of all used a bottom‐up strategy to overcome this boundary, and we approached individual patients with a specific interest in the topic via social media or (informal) patient meetings. We asked moderators of targeted Facebook groups (for lung disease and depression) if we could use their platform, and liaised with medical specialists to announce our research project and provide our contact information for those interested in receiving further information. In addition, events announced on websites of patient organizations were attended by research team members. After gaining the explicit permission of the organizers of such events, we distributed flyers or approached patients (for cardiovascular diseases and diabetes). Only patients with rheumatic disorders were recruited directly via the patient organization, since this patient organization has a very committed body of patients and the organization did acknowledge the importance of oral health(care) problems for its members.

Later on, in the consultation phase, it was found that patients were more than willing to help and they indicated that oral health care is an important topic. This was shown by the participants in the focus groups, who listed ‘contribute to research’ as the main reason to participate, in addition to gaining knowledge. One participant explained: ‘My motivation was to make a positive contribution to science and you always learn something from exchanging experiences’ *(focus group, patient 11)*. In addition, it was shown by the enormous response of 1495 participants to the survey distributed among patients that oral health care is indeed a topic that concerns patients, even though patient organizations are not aware of their patients' interest in this topic. Establishing a shared research agenda helped to generally expose the patients' interest in oral health care, but more specifically, it helped to alert practitioners and the patient organizations to this interest.

A third boundary was related to the *diversity of oral health‐care patients*. Although everyone qualifies as an oral health‐care patient, experiences might diverge significantly across the high‐risk patients' groups on which our study focused. We expected that patients with different types of chronic diseases would ensure that their perspective was heard during the focus groups and thus protect their boundaries, making boundary crossing difficult or even impossible. Therefore, we consulted patients within each chronic disease group separately. We anticipated that recognition of daily problems related to oral health care among patients with a similar medical background would stimulate discussions and avoid conflicts during the focus groups. Although there were no conflicts, it became clear that patients with the same disease encounter a range of problems in their daily life related to oral health care. Therefore, many topics discussed during the focus groups were largely related to problems concerning patients' individual situation. As a participant from one of the focus groups explained: ‘There are so many types of rheumatism, and you can never actually say something that applies to everyone… So, that's why I say you won't find consensus among all patients with rheumatic diseases’ *(focus group, patient 2)*. Although patients faced a variety of problems in their daily life, our approach stimulated them to consider each other's perspective and find common denominators; that is, boundary crossing was facilitated by the research agenda setting process. The interviews after the focus groups revealed that a moderated discussion of the various individual (oral) health‐care problems fostered learning from each other. One participant explained how he had learned about sleep apnoea: ‘Especially about sleep apnoea (I learned from other participants about), a topic I also came into contact with when among heart patients, but I was surprised how often this occurs… I found that informative, that it is actually so often present’ *(focus group, patient 10)*.

### 3.3 Boundaries between patients and practitioners

Two main boundaries between patients and practitioners were identified and can be described as follows: (a) the differences in perspectives and interests of patients and practitioners, and (b) the uncertainty about the value of patient involvement.

In line with the Dialogue Model^16^ and based on the expected heterogeneity between stakeholders,^6^ we first consulted each actor group separately to address *the differences in perspectives and interests of patients and practitioners*. These differences were reflected in the data collected during the exploration and consultation phase. The topics supplied by practitioners mainly focused on treatment decisions, while the impact of oral health(care) on patients' daily life was rarely considered. Patients expressed different ideas and needs about oral health(care), specifically concerning their experiences in daily life. Patients often doubted whether the decisions practitioners made were in their best interests. One patient explained: ‘I am very positive [about patient involvement]; [it is] useful that practitioners are confronted with the needs of the patients – they don't take that sufficiently into account’ *(focus group, patient 8)*. After the consultation and the prioritization of topics, a consensus meeting was organized to create the shared research agenda. Following this two‐step procedure ensured that the topics on the final research agenda were of interest to both patients and practitioners. This procedure was needed to create mutual appreciation and establish an equitable discussion.

Moreover, both patients and practitioners expressed *uncertainty about the value of patient involvement*. During the first stakeholder meeting in the exploration phase, an oral health‐care professional noted: ‘If the priorities of patients and practitioners do not match, the professional should have the final say’. One patient also expressed uncertainty about what to expect of the focus group: ‘My goal was to contribute of course, and I didn't know what else to expect. At first, I thought the researchers and discussion moderator would take the initiative, but we [the patients] were in the lead and it was all about us’ *(focus group, participant 9)*. Because the value of patient involvement was unclear to both practitioners as well as patients, it was decided to gradually increase the role of patients in the project.

During each stakeholder meeting, the focus on patient involvement was gradually emphasized. This was reflected by the number of patient representatives attending the stakeholder meetings, which increased from one during the first meeting to four during the third meeting. Accordingly, the number of topics concerning patient involvement that were discussed during the stakeholder meetings increased too. In addition, all meetings were moderated so that patient input was secured and valued.

At the end of the project, topics identified by patients and by practitioners separately were discussed among groups during the consensus meeting. The design of the consensus meeting ensured that patients and practitioners were equally represented and given sufficient room to present their respective perspective in a safe environment and an open atmosphere. The discussions during this meeting were facilitated carefully. The research agenda‐setting process made patients and practitioners realize that experiential knowledge from patients is a valuable source of information in research agenda setting.

### 3.4 Boundaries in relation to practitioners

Three boundaries for practitioners were identified: (a) the lack of familiarity of oral health‐care practitioners with research agenda setting, (b) the existence of a lack of urgency for a research agenda and (c) practitioners prefer topics that fit their own specialty.

Firstly, we expected a boundary that was created by the *lack of familiarity of oral health‐care practitioners with research agenda setting*. During all stakeholder meetings, this was brought to our attention by the participants. It was not only the lack of familiarity of individual oral health‐care practitioners that shaped this boundary but also the lack of interest of professional oral health‐care organizations. The response rate of practitioners to the first survey was initially low; it took nearly 8 months to attract a substantial number of respondents and collecting topic suggestions was therefore more time‐consuming than anticipated. A similar response rate to the second survey was reached in less time (3 months). Since the same outreach strategies were used to reach practitioners who wanted to participate, we interpreted this difference as being the result of fostering the involvement of practitioners in the agenda‐setting process.

Secondly, related to the first practitioner boundary, there was a *lack of urgency for a research agenda* among practitioners. They felt that there was no need for change and were satisfied with their current way of working. During the first stakeholder meeting, this was brought to attention of the researchers: ‘One of the hurdles will be the lack of urgency for a research agenda. The feeling exists that things are fine the way they currently are’. Therefore, the emphasis in communication about the project (in professional media) was placed on the profits for both the individual professional and the profession. Setting the research agenda created an awareness that increased evidence was needed to improve the quality of oral health care.

A final boundary involved the expectation that *practitioners within oral health care prefer topics that fit their own specialty*. For example, we expected practitioners with specific interest in endodontics to prioritize topics in this research area. However, to develop a broad research agenda we challenged oral health‐care practitioners to also consider topics beyond their expertise. We implemented this in the design of the second survey that was distributed among practitioners in the following way: after the collection of suggestions of topics for future oral health‐care research via a first survey, a second survey study was conducted in which oral health‐care practitioners prioritized research topics based on the collected subjects. The research topics were categorized into ten research domains. The practitioners could only select a maximum of two topics within a research domain. In this way, a constraint was placed and practitioners had to prioritize topics covering all domains of oral health care. This stimulated them to broaden their focus and to reflect on uncertainties in daily practice that might be relevant to a larger and more diverse group of oral health‐care practitioners.

## 4 DISCUSSION

In the last decade, involving patients in research agenda setting is gradually becoming more common. However, health‐care domains with a wide range of diverse patients who are poorly organized, such as oral health care, may lag behind.^7^, ^19^ In the Netherlands, a research agenda for oral health care was established with input from oral health‐care patients and practitioners. This project applied the boundary‐work theory.^20^, ^21^ In order to gain an understanding of the boundaries that were encountered and crossed in the process of setting a research agenda, this case study was successfully used as a boundary object. This study showed that the research agenda itself might function as a boundary object to cross boundaries between patient groups and between patients and practitioners. Using the Dialogue Model in setting the research agenda helped to overcome and transform expulsion and protection boundaries into mutual appreciation, via expansion boundary work. The concept of boundary object was useful with respect to this. In the process of setting the research agenda, expansion boundary work was established by carefully crossing boundaries between patient groups and between patients and professionals. Reflexive learning made a significant contribution to this boundary crossing by enabling groups to gain insight into each other's underlying perspectives. In this way, boundaries, which seemed problematic initially, were translated into productive processes and outcomes via the use of a boundary object.

It should be recognized that most topics of the shared research agenda are related to preventative care and the health‐care system rather than curative care and treatments, which is in line with the contemporary shift in focus in oral health care.^34^ This indicates a broader view of oral health care and oral health and reflects the interests of both patients and oral health‐care practitioners.

Regarding general boundaries, boundaries that were found for patients as well as practitioners, we have encountered the difficulty of obtaining the support of a diverse group of stakeholders. Lack of support on institutional level (ie the urgency of oral health as an important research topic is not supported on an organizational policy level) was specified by patients' boundaries (lack of interest in the topic by patient organizations despite worldwide ranking in burden of diseases) and boundaries of practitioners (lack of urgency for research agenda). To deal with these boundaries, multiple boundary crossing strategies were needed, such as scheduling regular stakeholder meetings, promoting the project and using many recruitment strategies to include a wide range of oral health‐care patients and practitioners. However, we did not bring together officials of patient organization and professional organization to engage in boundary crossing. Therefore, the boundary may still exist at the institutional or organizational policy level. We considered the equal representation and support of patients and practitioners to be essential to establish a shared research agenda representing topics that are important to both patients and practitioners.

In relation to patient boundaries, setting the research agenda helped to cross multiple boundaries. One of the major boundaries for patient involvement is not knowing who the target group is and how they should be approached.^17^ We dealt with this boundary first by focusing on patients with diseases that carry a high‐risk for poor oral health and second by considering a variety of recruitment strategies. During the focus groups, it was shown that within specific patient groups that included people who were suffering from the same disease, a wide variety of oral health‐care problems were encountered. However, proposing these topics to a wider range of individuals in the survey study showed that these topics did not only apply to the consulted patient groups. Consulting patients via this survey confirmed the diversity of oral health‐care patients and the needs of different types of patients.^6^, ^10^ Our approach ensured that the individual voice of high‐risk patients was transformed into a voice representing a larger patient group.^31^ In line with previous studies,^12^ we found that patients felt empowered by participation.^35^

Ultimately, the approach to setting the research agenda helped to overcome existing boundaries among practitioners. It encouraged a broad range of practitioners to reflect on uncertainties in daily practice which broadened their focus and shaped their awareness of the greater need for evidence in oral health care. As a result, this research agenda reflects the interests both of practitioners and of patients.

We acknowledged that dynamics between insider‐outsider groups (patients versus practitioners) are shaped by multiple boundaries rather than by one single boundary,^36^, ^37^ and we therefore decided not to address single boundaries in isolation from each other. To illustrate this point, the research agenda setting served as a boundary object that unites the perspectives of patients and practitioners. To achieve this, it was essential to involve the practitioners at an early stage and to gradually increase the patients' role. At the start, it was unclear how to value patient involvement. Previous research^17^ has shown the existence of boundaries regarding patients' involvement in policy and in the relevant guidelines for its implementation. It is therefore important to emphasize the role of the patients from the start and to increase their role gradually. The patients and practitioners realized and acknowledged the value of experiential knowledge of patients. This is essential for the success of the research agenda, as dismissing the input of patients will lead to the exclusion of patients.^7^

Hence, our study showed that to unite perspectives of patients and practitioners, it is essential to be aware of possible boundaries that might be encountered to respond adequately. Our study acknowledged that boundary work is a process, dispersed and also political.^36^ Identifying and addressing boundaries is a time‐consuming process: it is necessary to give stakeholders enough time to become familiar with research agenda setting and each other's perspectives. To accomplish this, the underlying principles of the Dialogue Model—such as enclave deliberation, reflexive learning and an emergent design—seemed particularly appropriate. Boundaries in our study mostly occurred because patients as well as practitioners were unfamiliar with setting a research agenda and lacked knowledge about the process and each other's viewpoints. Structuring patient involvement according to the Dialogue Model—and acknowledging and acting on the different perspectives of the involved stakeholders—helped to overcome these boundaries, as the strategies in this model are on a rational level. In contrast, when boundaries are grounded in emotions rather than lack of knowledge or familiarity, the Dialogue Model might not be able to solve these boundaries.^19^ This is in line with ideas put forward by Star & Griesemer^30^ about boundary objects. Although the concept of boundary objects can be useful to cross boundaries and create a shared reality, these boundary objects are always context dependent. Boundary objects are one way to deal with conflicting perspectives. In other situations, other approaches, such as fragmentation (ie breaking into smaller groups rather than uniting groups^38^ ), might be more suitable. It is, therefore, essential to be reflective and anticipate the type of boundaries that might occur and why before deciding upon a particular strategy. Boundary objects are not a guaranteed solution for overcoming boundaries.

### 4.1 Strengths, limitations and future research

In this study, we chose a qualitative process evaluation approach based on a multi‐stakeholder perspective. While this approach might have limited the transferability of our results, our study provided unique and diverse insights into the boundaries encountered during the process of research agenda setting in relation to oral health care, using boundary‐work theory. This way, the chosen evaluation approach helped us to deepen our understanding of how the Dialogue Model served to expand and cross boundaries. We have included a variety of oral health‐care patients and practitioners. The use of the structure of the Dialogue Model helped to gain support from the practitioners and the patients during all phases of the agenda‐setting process. Setting the research agenda helped to enhance the value of patient involvement and created patient empowerment and shared ownership of the research agenda.

This study only reports on the boundaries of two relevant stakeholder groups, patients and practitioners that are often ignored in setting a research agenda. Still, other stakeholders, such as policymakers, insurance companies and researchers, might have introduced additional boundaries. Moreover, this study only reports on boundaries for the research agenda setting. Therefore, the boundaries which might arise during programming and implementation of this research agenda remain unknown.

Previous research^1^ has shown that patient involvement is often not continued in these subsequent phases. When this research agenda is taken further into programming and implementation, the boundary‐work theory and Dialogue Model may also serve to maintain the involvement of a diverse group of patients and practitioners and unite their perspectives and priorities with those of other relevant stakeholders.

## 5 CONCLUSION

The established oral health‐care research agenda was endorsed by both patients and practitioners. This case study showed that setting the research agenda using (the principles of) the Dialogue Model contributed to elucidating boundaries within and between groups of patients and practitioners in the field of oral health care. Structuring patient involvement according to the Dialogue Model enabled patients and practitioners to safely cross boundaries that emerged during different phases of patient involvement in this case study. The principles of reflexive learning, neutral process facilitation and using an emergent design seemed to be particularly valuable.

## CONFLICT OF INTEREST

The authors declare that there is no conflict of interest.

## Data Availability

The anonymized data (in Dutch) that support the findings of this study are available from the corresponding author upon reasonable request.

## APPENDIX A

### Questionnaire items

1. The number of oral health‐care practitioners and patient (representatives) was well balanced.
2. In my experience I adequately represented my stakeholder group.
3. The goal of the consensus meeting was clear to me.
4. I knew what was expected of me during the meeting.
5. There was sufficient time to share my ideas during the meeting.
6. The supplied information and presentations were easy to follow and understand.
7. I felt taken seriously by other participants.
8. I felt involved in the discussions during the meeting.
9. The atmosphere at the meeting was pleasant.
10. I felt free to give input during the meeting.
11. I felt like everyone was given equal opportunity to give input.
12. I was actively asked for my opinion/vision.
13. I could give a valuable contribution.
14. I found the contribution of others valuable.
15. A dialogue between patients and oral health‐care practitioners is a useful method to establish a shared research agenda.
16. I was satisfied with the procedure of the meeting.
17. As a participant of this meeting, I gained insight into the perspectives of other participants.
